# Novel Synthetic Oxazines Target NF-κB in Colon Cancer *In Vitro* and Inflammatory Bowel Disease *In Vivo*

**DOI:** 10.1371/journal.pone.0163209

**Published:** 2016-09-29

**Authors:** Anilkumar C. Nirvanappa, Chakrabhavi Dhananjaya Mohan, Shobith Rangappa, Hanumappa Ananda, Alexey Yu Sukhorukov, Muthu K. Shanmugam, Mahalingam S. Sundaram, Siddaiah Chandra Nayaka, Kesturu S. Girish, Arunachalam Chinnathambi, M. E. Zayed, Sulaiman Ali Alharbi, Gautam Sethi, Basappa, Kanchugarakoppal S. Rangappa

**Affiliations:** 1 Laboratory of Chemical Biology, Department of Chemistry, Bangalore University, Central College campus, Bangalore-560001, India; 2 Department of Studies in Chemistry, University of Mysore, Manasagangotri, Mysore-570005, India; 3 Department of Studies in Molecular Biology, University of Mysore, Manasagangotri, Mysore-570005, India; 4 Frontier Research Center for Post-Genome Science and Technology, Hokkaido University, Sapporo 0600808, Japan; 5 N.D. Zelinsky Institute of Organic Chemistry, Leninsky Prospect, 47, Moscow 119991, Russia; 6 Department of Pharmacology, Yong Loo Lin School of Medicine, National University of Singapore, 117 597, Singapore, Singapore; 7 Department of Studies in Biochemistry, University of Mysore, Manasagangotri, Mysore-570005, India; 8 Department of Botany and Microbiology, College of Science, King Saud University, Riyadh -11451, Kingdom of Saudi Arabia; University of Kansas School of Medicine, UNITED STATES

## Abstract

Aberrant activation of nuclear factor kappa B (NF-κB) has been linked with the pathogenesis of several proinflammatory diseases including number of cancers and inflammatory bowel diseases. In the present work, we evaluated the anticancer activity of 1,2-oxazines derivatives against colorectal cancer cell lines and identified 2-((2-acetyl-6,6-dimethyl-4-phenyl-5,6-dihydro-2H-1,2-oxazin-3-yl)methyl)isoindoline-1,3-dione (API) as the lead anticancer agent among the tested compounds. The apoptosis inducing effect of API was demonstrated using flow cytometry analysis and measuring the caspase 3/7 activity in API treated cells. Based on the literature on inhibition of NF-κB by oxazines, we evaluated the effect of 1,2-oxazines against the ability of NF-κB binding to DNA, NF-κB-dependent luciferase expression and IκBα phosphorylation. We found that, API abrogate constitutive activation of NF-κB and inhibits IκBα phosphorylation in HCT116 cells. Our *in silico* analysis revealed the binding of oxazines to the hydrophobic cavity that present between the interface of p65 and IκBα. Given the relevance with aberrant activation of NF-κB in inflammation bowel disease (IBD), we evaluated the effect of API on dextran sulphate sodium-induced IBD mice model. The treatment of IBD induced mice with API decreased the myeloperoxidase activity in colonic extract, modulated the colon length and serum levels of pro- and anti-inflammatory cytokines such as TNF-α, IFN-γ, IL-6, IL-1β and IL-10. Furthermore, the histological analysis revealed the restoration of the distorted cryptic epithelial structure of colon in the API treated animals. In conclusion, we comprehensively validated the NF-κB inhibitory efficacy of API that targets NF-κB in *in vitro* colon cancer and an *in vivo* inflammatory bowel disease model.

## Introduction

Ulcerative colitis and Crohn’s disease are the disorders of the gastrointestinal tract and leading types of inflammatory bowel disease (IBD) caused by various environmental and genetic factors [1]. IBD is characterized by recurrent inflammation of intestine due to transmural infiltration of immune cells such as macrophages, mast cells and lymphocytes leading to disruption of mucosa and ultimately ulceration [2]. Patients suffering from IBD are with the high risk of development of colorectal cancer [3,4]. Currently, sulfasalazine (5-aminosalicylic acid derivatives), corticosteroids and several nonsteroidal anti-inflammatory drugs (NSAIDs) have been used to treat IBD [5]. However, these drugs have limited efficacy and the long term use is associated with multiple adverse effects [6]. Therefore, it projects the need of identifying a chemically novel, biologically active therapeutic agents with minimal adverse effects.

Nuclear factor kappa B (NF-κB) is a proinflammatory transcription factor resides abundantly in the cytoplasmic compartment of most mammalian cells and aberrant expression has been linked in the pathogenesis of IBD [7]. NF-κB family comprises of five types of subunits namely, p50 (NF-κB1), p52 (NF-κB2), p65 (RelA), RelB and c-Rel [8]. It is present either in homodimeric or heterodimeric form in association with its negative regulator, IκB (inhibitory κB) and IκB prevents translocation into nucleus, in turn modulates the expression of NF-κB targeted genes [9]. The signaling by various ligands ubiquitylates the IκB and subject it to proteasome mediated degradation and thereby NF-κB translocate into nucleus [10]. NF-κB is known to modulate the expression of over 500 genes involved in multiple cellular events such as cell transformation, proliferation (IL-1β, cyclin D1), anti-apoptosis (Bcl-2, Bcl-xL), immortality (telomerase), survival (cIAP, xIAP), inflammation (TNF, IL-1), angiogenesis (VEGF, IL-8), invasion (uPA, MMPs), and metastasis (ICAM-1, CXCR-4) [11]. Besides, the deregulation of NF-κB is tangled with initiation, progression and maintenance of several proinflammatory diseases including cancers, inflammatory bowel disease, arthritis, and asthma.

Several derivatives of oxazine have been studied comprehensively in various cancer models and reported to possess very good antitumor potential [12–14]. Multiple reports have suggested that oxazines are good inhibitors of NF- κB signaling pathway [15,16]. Specifically, γ- and δ-tocotrienol conjugated oxazine derivatives displayed decrease in the levels of phosphorylated NF-κB syngeneic +SA mammary tumors [15]. 2-ethoxy-4,5-diphenyl-1,3-oxazine-6-one is a small molecule reported to decrease the levels of nuclear NF-κB in lipopolysaccharide (LPS)-induced NGF-differentiated PC12 cells [16]. Given the relevance with aforementioned reports and in continuation of our effort to synthesize various heterocycles to explore their medicinal properties [17–24], herein we prepared the series of oxazine derivatives and evaluated their effect on HCT116 cells. The lead compounds 2-((2-acetyl-6,6-dimethyl-4-phenyl-5,6-dihydro-2H-1,2-oxazin-3-yl)methyl)isoindoline-1,3-dione (API) and dimethyl 2-((2-acetyl-4-(4-methoxyphenyl)-6,6-dimethyl-5,6-dihydro-2H-1,2-oxazin-3-yl)methyl)malonate (DMO) were chosen and evaluated for their NF-κB DNA binding inhibitory activity and NF-κB dependent luciferase expression studies. Finally, in vivo anti-inflammatory activity of the API was reported using dextran sulfate sodium (DSS) induced IBD mouse model.

## Materials and Methods

### Animals, cell lines and reagents

All animal experiments were approved by the Institutional Animal Ethical Committee, Department of Studies in Zoology, University of Mysore, Mysore and were in accordance with the guidelines of the Committee for the Purpose of Control and Supervision of Experiments on Animals (CPCSEA). HCT116 (colorectal cancer) and LO2 (immortal hepatic) cell lines were initially purchased from ATCC and were cultured in DMEM medium containing 10% fetal bovine serum, 1mM L-glutamine, 1 mM sodium pyruvate, antibiotic and antimycotic agent. Human Colonic epithelial cells (CoEpic) were purchased from Science Cell Research Laboratories (Carlsbad, CA, USA). Colonic epithelial cell culture medium and supplement were purchased from Science Cell Research Laboratories (Carlsbad, CA, USA). The cells were cultured in poly-l-lysine coated culture flasks in colonic epithelial cell medium containing supplement. Caspase 3/7 assay kit was purchased from Promega Inc (Hercules, CA). Dextran sulfate sodium salt (DSS, MW 36–50 kDa) was purchased from MP Biomedicals, Solon, USA. Etacept was obtained from CIPLA, Mumbai, India. Sulfasalazine (SZ) was from Cadila Healthcare Ltd., Ahmedabad, India. Murine mini ELISA development kits for TNF-α, IFN-γ, IL-6, IL-1β, and IL-10 were purchased from PeproTech, KHC Healthcare, New Delhi, India. IκBα and GAPDH antibodies were obtained from Santa Cruz Biotechnology (Santa Cruz, CA). Antibodies against phospho-specific IκBα (Ser 32), was purchased from Cell Signaling Technology (Beverly, MA). Nuclear extraction and DNA binding kits were obtained from Active Motif (Carlsbad, CA). All other chemicals were of analytical grade and were purchased from Sisco research laboratories, Mumbai, India.

### Synthesis of 1,2-oxazines

1,2-oxazines (**1a-f**) were synthesized as described previously, whose ^1^H and ^13^C NMR spectra are consistent with literature data and showed more than 95% purity [25]. The 1,2-oxazines (**1g-i** and **2a-c)** were prepared according to literature protocol [26] and their complete characterization was reported recently [27]. The library of all the tested 1,2-oxazine structures are given in Table 1.

**Table 1:** 

Entry	Title Compounds	Cytotoxicity	CDOCKER Energy (-CE)	CDOCKER interaction energy (-CIE)
**1a**	(S)-2-((6,6-dimethyl-4-phenyl-5,6-dihydro-4H-1,2-oxazin-3-yl)methyl)isoindoline-1,3-dione	36.7	-126.725	-27.441
**1b**	Dimethyl (S)-2-((4-(4-methoxyphenyl)-6,6-dimethyl-5,6-dihydro-4H-1,2-oxazin-3-yl)methyl)malonate	NA	-49.046	4.201
**1c**	Dimethyl 2-(((4S)-4-(4-methoxyphenyl)-4a,5,6,7,8,8a-hexahydro-4H-benzo[e][1,2]oxazin-3-yl)methyl)malonate	36.2	-66.919	-8.642
**1d**	Dimethyl 2-(((4S)-4-phenyl-4a,5,6,7,8,8a-hexahydro-4H-benzo[e][1,2]oxazin-3-yl)methyl)malonate	32.7	-70.946	-18.931
**1e**	Dimethyl 2-(((4S,5R,8S)-4-phenyl-4a,5,6,7,8,8a-hexahydro-4H-5,8-methanobenzo[e][1,2]oxazin-3-yl)methyl)malonate	30.1	-189.683	-56.764
**1f**	Dimethyl (R)-2-((4,6,6-trimethyl-5,6-dihydro-4H-1,2-oxazin-3-yl)methyl)malonate	NA	11.307	24.401
**1g**	Methyl (S)-2-(4-(4-methoxyphenyl)-6,6-dimethyl-5,6-dihydro-4H-1,2-oxazin-3-yl)acetate	42.1	-21.13	6.733
**1h**	Methyl 2-((4S)-4-phenyl-4a,5,6,7,8,8a-hexahydro-4H-benzo[e][1,2]oxazin-3-yl)acetate	NA	10.342	20.658
**1i**	Methyl 2-((4S,5R,8S)-4-phenyl-4a,5,6,7,8,8a-hexahydro-4H-5,8-methanobenzo[e][1,2]oxazin-3-yl)acetate	14.3	-96.932	-8.225
**2a (API)**	2-((2-Acetyl-6,6-dimethyl-4-phenyl-5,6-dihydro-2H-1,2-oxazin-3-yl)methyl)isoindoline-1,3-dione	6.2	-330.737	-103.675
**2b (DMO)**	Dimethyl 2-((2-acetyl-4-(4-methoxyphenyl)-6,6-dimethyl-5,6-dihydro-2H-1,2-oxazin-3-yl)methyl)malonate	6.5	-211.836	-52.072
**2c**	Dimethyl 2-((2-acetyl-6,6-dimethyl-4-phenyl-5,6-dihydro-2H-1,2-oxazin-3-yl)methyl)malonate	18.3	-421.525	-68.115
**BPO**	Phenyl(4-phenylcyclopenta[c][1,2]oxazin-7-yl)methanone		-200.245	-58.732

### MTT Assay

The cytotoxic effect of 1,2-oxazines against HCT116 cells was determined by the MTT dye uptake method as described previously [28,29]. Briefly, the cells (2.5×10^4^/ml) were incubated in triplicate in a 96-well plate in the presence or absence of the indicated concentrations of compounds in a final volume of 0.2 ml for different time intervals at 37°C. Thereafter, 20 μl of MTT solution (5 mg/ml in PBS) was added to each well. After a 2 h incubation at 37°C, 0.1 ml of lysis buffer (20% SDS, 50% dimethylformamide) was added, incubation was done for 1 h at 37°C, and subsequently the optical density at 570 nm was measured by a Varioskan plate reader. Images of cell proliferation were also captured using a light microscope (Magnification 4x).

### Caspase 3/7 assay for apoptosis detection

Apoptosis was determined using caspase3/7 assay kit according to manufacturer’s protocol (Promega Inc, USA). HCT116 cells were incubated with or without API (5 or 10 μM) for 48 h. After incubation the cells were collected and assayed for caspase 3/7 activity.

### Flow cytometric analysis

The effect of API on cell cycle of HCT116 cells was performed as described previously [30]. To determine the effect of API on the cell cycle, cells were treated with API at indicated doses up to 48 h. Thereafter cells were washed, fixed with 70% ethanol, and incubated for 30 min at 37°C with 0.1% RNase A in PBS. Cells were then washed again, resuspended, and stained in PBS containing 25 μg/ml propidium iodide (PI) for 30 min at room temperature. Cell distribution across the cell cycle was analyzed with a BD FACSVerse flow cytometer.

### NF-κB DNA binding assay

To determine NF-κB activation, we performed DNA binding assay using TransAM NF-κB Kit according to the manufacturer’s instructions and as previously described [31]. Briefly, 20 μg of nuclear proteins was added into 96-well plate coated with an unlabeled oligonucleotide containing the consensus binding site for NF-κB (5′-GGGACTTTCC-3′) and incubated for 1 h. The wells were washed and incubated with antibodies against NF-κB p65 subunit. An HRP conjugated secondary antibody was then applied to detect the bound primary antibody and provided the basis for colorimetric quantification. The enzymatic product was measured at 450 nm by microplate reader (Tecan Systems).

### NF-κB luciferase reporter assay

The effect of DMO and API on constitutive a NF-κB-dependent reporter gene transcription in HCT116 cells was determined as previously described [32]. NF-κB responsive elements linked to a luciferase reporter gene were transfected with wild-type or dominant-negative IκB. The transfected cells were then treated with various doses of API or DMO for 6 h. Luciferase activity was measured with a Tecan (Durham, NC, USA) plate reader and normalized to β-galactosidase activity. All luciferase experiments were done in triplicate and repeated twice.

### Western blotting

Western blotting analysis was performed as described earlier [33,34]. For detection of phospho-proteins, cytoplasmic extracts of API treated cells were prepared. Lysates were then spun at 14,000 rpm for 10 min to remove insoluble material and resolved on SDS gel. After electrophoresis, the proteins were electrotransferred to a nitrocellulose membrane, blocked with 5% non-fat milk, and probed with anti-phospho-IκBα/IκBα antibodies overnight at 4°C. The blot was washed, exposed to HRP-conjugated secondary antibodies for 1 h, and finally examined by chemiluminescence.

### In vivo anti-inflammatory studies

The *in vivo* anti-inflammatory efficacy was evaluated as described earlier [5]. Briefly, adult Swiss albino mice (25–30 g) were injected intraperitoneally with 2 ml of 3% thioglycolate (TG) broth or sterile saline. After 10 min, API (5, 10 or 15 mg/kg body weight) in saline was injected through a lateral tail vein. After 24 h, LPS (1.0 μg) was injected intraperitoneally, and 1 h later, the peritoneal cavities were lavaged with 4 ml of PBS containing 3 mM EDTA and total number of inflammatory cells was counted. Heparin (10 mg/kg) was used as a positive control.

### Molecular docking study

Accelrys Discovery Studio (DS) version 2.5 was used for molecular modeling studies [18]. The crystal structure of IκBα/NF-κB complex (PDB: 1IKN) was considered and prepared the proteins for molecular docking studies using the protein preparation modules of DS. The sdf format of the ligands were prepared in parallel and the CDOCKER (CHARMm-based DOCKER) of DS was performed. The 1,2-oxazine ligands were docked at the interface of p65 (a subunit of NF-κB) and IκBα. The CDOCKER energy, and the CDOCKER interaction energy was tabulated and ligand bound NF-κB complex was visualized using DS visualization tool.

### DSS-induced inflammatory bowel disease (IBD) model

Swiss albino mice (6–8 weeks old) weighing 22–25 g were used in the present study and were fed with standard mice diet and given free access to reverse osmosis (RO) water. Animals were acclimatized for 10 days before start of the experiment. Animals were randomly divided into 5 groups and each consisted of 6 mice; group 1- RO water control, group 2- DSS induced, group 3- DSS induced mice treated with SZ (500 mg/kg/day, oral gavage), group 4- DSS induced mice treated with Etacept (5 mg/kg/day, subcutaneously injected), group 5- DSS induced mice treated with API (5 mg/kg/day, intraperitoneal injection). Etacept was used as standard TNF-α inhibitor and SZ as standard therapeutic drug against colitis. IBD was induced to all groups except group 1 by administering 5% DSS (w/v) in RO drinking water for 4 days. Respective treatment was given from 5^th^ day up to 9^th^ day. Animals were anesthetized using sodium pentobarbital (30 mg/kg body weight, intraperitoneal injection) to minimize the suffering of and were sacrificed on 10^th^ day by cardiac puncture and blood was collected, serum was separated and stored at -20°C to assess various cytokines. Further, the colon was excised from the experimental animals, flushed with ice-cold PBS and processed for histological analysis. The experimental animals were monitored every 12 h upon DSS treatment and body weight, DAI were recorded every 24 h until the completion of the experiment. There was no mortality in any groups of mice used in the present study.

### Disease activity index (DAI)

DAI was scored as described earlier by Cooper *et al* [35]. In brief, weight loss, stool consistency, and gross bleeding are the three individual parameters considered to assign the scoring of DAI. Stool consistency scored zero for normal stool; two for loose stool; and four for diarrhea. Rectal bleeding scored zero for normal; two for occult bleeding; and four for gross bleeding. Lastly, severity of colitis in the colons were analyzed by measuring the length of the colon, which is an indirect evidence of colonic inflammation.

### Myeloperoxidase (MPO) assay

The excised colonic tissue from all the groups were homogenized in 50 mM potassium phosphate buffer pH 6.0 containing 0.5% hexadecyltrimethyl ammonium bromide. Tissue debris were removed by centrifuging the homogenates at 8,000 rpm for 4 min at 4°C. Supernatant (10 μL) was taken in 96-well plate in triplicate, and 200 μL of ODA-H_2_O_2_reagent (0.167 mg/mL *O*-dianisidinedihydrochloride and 0.05% H_2_O_2_) was added to each well including the well containing 10 μL of buffer alone, which served as blank. Absorbance was measured at 450 nm using multimode plate reader (Varioskan, Thermo Scientific) at 0, 30, and 60 S. The difference between two time points was taken, and the MPO activity was calculated (MPO constant: 1.13 ×10^−2^).

### Estimation of serum cytokines

The serum levels of pro-inflammatory (TNF-α, IFN-γ, IL-6, and IL-1β) and anti-inflammatory (IL-10) cytokines were estimated using ELISA kits according to the manufacturer’s protocol.

### Histological analysis

Colonic tissues were fixed overnight in 10% phosphate-buffered formalin and were dehydrated using alcohol and chloroform mixture. The processed tissues were embedded in paraffin wax, and 5 μm thick sections were prepared. Further, the sections were stained with hematoxylin-eosin dye (H & E) and photographed under an Axio Imager A2 microscope (Zeiss, Oberkochen, Germany). The method of Gonzalez-Rey *et al*. was followed for histological scoring of inflammation of colon sections on a 0–3 graded scale [36]. Zero for no sign of inflammation; one for low leukocyte infiltration; two for moderate leukocyte infiltration, thickening of the colon, moderate goblet cell loss, and focal loss of crypts; and three for transmural infiltration, massive loss of goblet cells, and diffuse loss of crypts. Irrespective of the treatments, each slide was scored from five random spots.

### Statistical analysis

Results are expressed as mean ± SEM of three independent experiments. Statistical significance was determined using one-way ANOVA, followed by Bonferroni post-hoc test. Significance was accepted at *p* < 0.05 (*), *p* < 0.01 (**) and *p* < 0.001 (***). Data was analyzed using the statistical package GraphPad Prism (GraphPad Software 5.0). Student t-test was used to analyze NF-κB activation data. p<0.05 was considered statistical significant *p<0.05; **p<0.005.

## Results

### 1,2-Oxazine derivatives suppresses proliferation of colorectal cells in dose-dependent manner

**Fig 1:**
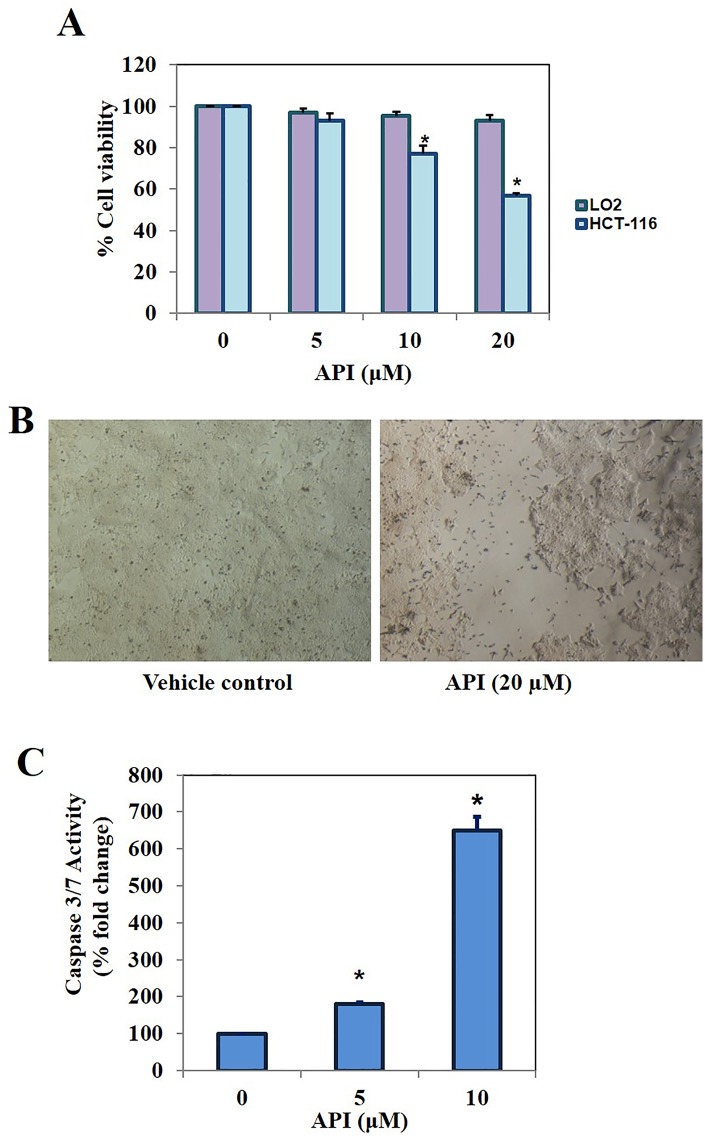
A. The lead compounds identified among the 1,2-oxazine derivatives tested decreased the cell proliferation of HCT116 cells in dose-dependent manner. B. Microscopic images to demonstrate the inhibition of cell proliferation (Magnification 4x). C. HCT116 cells were treated with API for 48 h and caspase 3/7 assay was performed. We observed a significant dose dependent increase in caspase 3/7 activity levels demonstrating the ability of API to induce apoptosis.

We initially evaluated the cytotoxic efficacy of the series of 1,2-oxazines against colorectal cancer (HCT116) cells using MTT assay. Among the tested oxazine derivatives, API was found to be the most effective antiproliferative compound followed by DMO. Paclitaxel was used as positive control. We observed the treatment of HCT116 cells with API at 20 μM for 48 h reduced the cell proliferation nearly by 50% (Fig 1A). We further investigated the effect of lead compound on cell proliferation at different concentrations (0, 5, 10 and 20 μM) for 48 h and found the substantial decrease in the proliferation of HCT116 cells in a dose-dependent manner. Interestingly, API had no significant effect on the viability of CoEpic cells (Fig 1A). We have provided the microscopic images to demonstrate the inhibition of HCT116 cell proliferation (Fig 1B). We further noted that when HCT116 cells were treated with API for 48 h, a significant dose dependent increase in caspase 3/7 activity levels were observed demonstrating its ability to induce substantial apoptosis (Fig 1C).

### API causes increased accumulation of HCT116 cells in Sub-G1 phase

**Fig 2:**
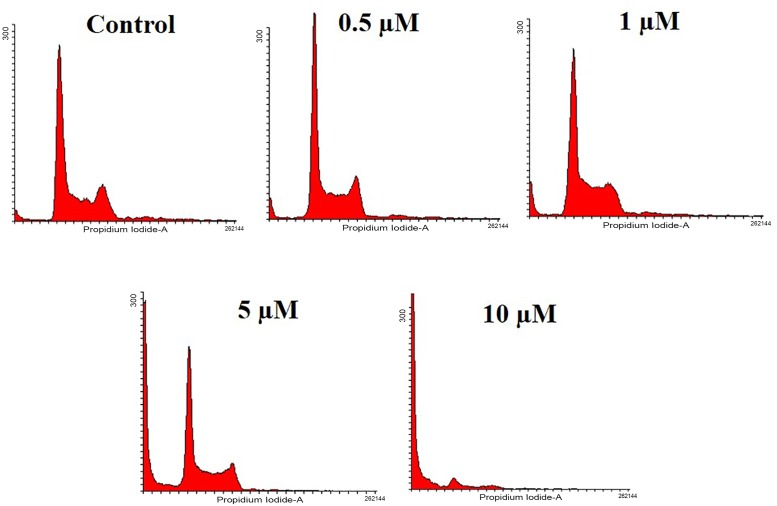
HCT116 cells were treated with different doses of API (0.5, 1, 5, and 10 μM) for 48 h, harvested and stained with propidium iodide and subjected to flow cytometry. Histogram obtained indicated the accumulation cells in sub-G1 phase.

The formation of hypodiploid cells due to the activation of caspase activated DNases, thereby fragmentation of nuclear DNA is a remarkable event in late apoptosis and the hypodiploid cells are detected as sub-G1 population [37]. Therefore, we investigated the effect of API on the distribution of cell cycle in HCT116 cells using propidium iodide staining. We observed the gradual increase in the sub-G1 phase population to nearly 80% at 10 μM concentration (Fig 2).

### API and DMO abrogate constitutive NF-κB activation in HCT116 cells

**Fig 3:**
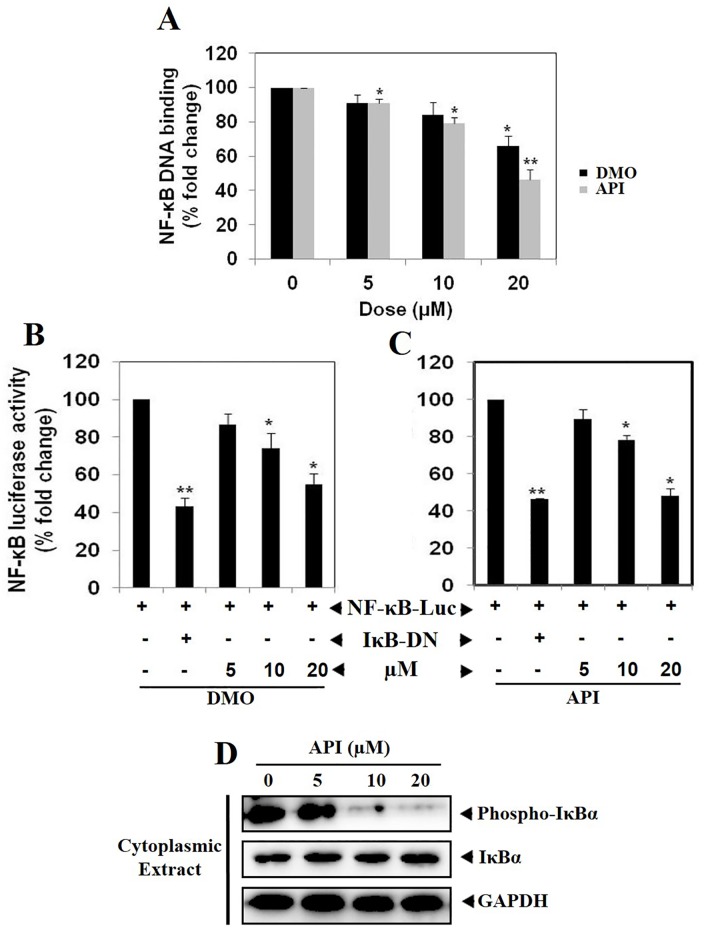
A. API and DMO suppresses NF-κB DNA binding ability in HCT116 cells. HCT116 cells were treated with DMO and API at indicated doses, nuclear extracts were prepared, and 20 μg of the nuclear extract protein was used for the ELISA-based DNA-binding assay *p<0.05; **p<0.005). B & C. NF-κB responsive elements linked to a luciferase reporter gene were transfected with wild-type or dominant-negative IκB and transfected cancer cells were treated at indicated doses for 6 h and luciferase activity was measured as described in Materials and Methods section. All luciferase experiments were done in triplicate and repeated twice (*p<0.05; **p<0.005). D. API abrogates constitutive IκBα phosphorylation in dose-dependent manner in HCT116 cells. HCT116 cells were treated with different concentrations of API (0, 5, 10 and 20 μM) for 6 h and cytoplasmic extract was prepared. Lysates were resolved on SDS gel and electrotransferred to a nitrocellulose membrane and probed with anti-phospho-IκBα/IκBα. The blot was washed, exposed to HRP-conjugated secondary antibodies for 1 h, and finally examined by chemiluminescence. GAPDH was used as loading control.

Oh and coworkers subjected 7,243 diverse compounds for their inhibitory efficacy against NF-κB signaling using a robust and reproducible high throughput screening TR-FRET Assay and identified 7-benzoyl-4-phenylcyclopenta [1,2] oxazine (BPO) as a potent inhibitor of IKKβ and in turn abrogates NF-κB signaling [38]. Therefore, we next investigated the effect of lead 1,2-oxazine derivatives, API and DMO on constitutive NF-κB activation in HCT116 cells. We found that treatment with various concentrations (0, 5, 10, 20 μM) of API and DMO suppressed constitutive NF-κB activity in a dose-dependent manner (Fig 3A). API reduced the NF-κB DNA binding ability by nearly 50% (**p<0.005) and these results indicate that API and DMO can modulate constitutive NF-κB activation in HCT116 cells. Although we observed that API and DMO inhibits NF-κB activation by NF-κB DNA binding assay, DNA binding alone does not always correlate with NF-κB-dependent gene transcription, indicating that additional regulatory steps may be involved in controlling NF-κB activation. To determine the effects of API and DMO on constitutive NF-κB-dependent reporter gene expression in HCT116 cells, transfection was performed as described in methods. In the presence of API and DMO NF-κB-dependent luciferase expression was inhibited in a dose-dependent manner with maximum inhibition of nearly 45% observed at 20 μM (Fig 3B and 3C). These results demonstrate that API and DMO can also abrogate constitutive NF-κB-dependent reporter gene expression.

### API abrogates constitutive IκBα phosphorylation in HCT116 cells

Because IκBα phosphorylation is essential for NF-κB activation, we next analyzed whether inhibition of NF-κB activation by API was due to inhibition of IκBα phosphorylation. We found that constitutive IκBα phosphorylation was suppressed in a dose dependent manner in cytoplasmic extracts obtained following the exposure of HCT116 cells to API for 6 h. Also, API treatment stabilized the levels of total IκBα in HCT116 cells (Fig 3D). This data indicates that API can also affect IκBα phosphorylation in colorectal cancer cells.

### In vivo anti-inflammatory studies

**Fig 4:**
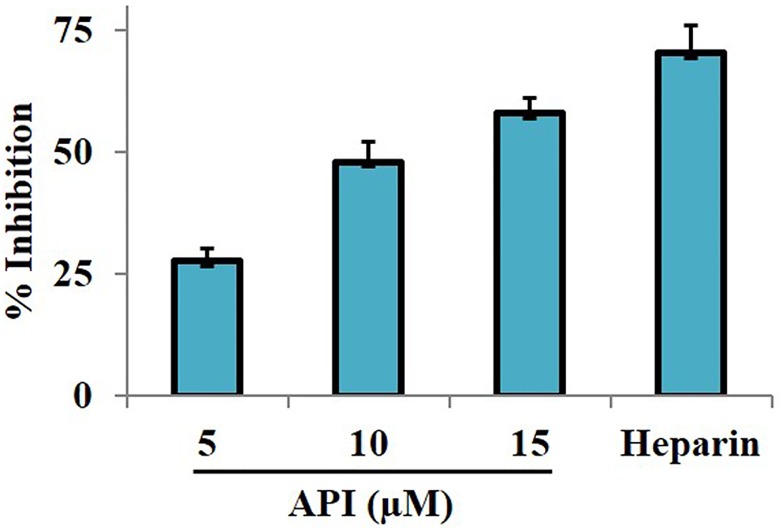
*In vivo* anti-inflammatory activity of API. The in vivo anti-inflammatory efficacy of API was evaluated by intraperitoneally administering thioglycolate broth and LPS to Swiss albino mice. The administration of API (5, 10, and 15 mg/kg body weight) suppressed the infiltration of macrophages into the peritoneal cavity. Heparin was used as positive control which inhibited the macrophage infiltration by 70%.

Previous findings have suggested that small molecules (including estradiol) abrogate LPS-induced TNF-α-mediated NF-κB activation which is a key event in inflammation [39]. Further, we investigated the effect of API on infiltration of macrophages to the peritoneal cavity of mice induced with thioglycolate broth and lipopolysaccharide. The results displayed decrease in infiltration of inflammatory cells in the lavage fluid by 27.6, 48, and 58% at 5, 10, and 15 mg/kg body weight respectively, compared with vehicle treated mice (Fig 4). Heparin suppressed the infiltration of inflammatory cells in the lavage fluid by 76% at 10 mg/kg body weight. These results indicate that, API imparts *in vivo* anti-inflammatory activity by modulating NF-κB signaling pathway.

### In silico interaction of 1,2-oxazines towards NF-κB

**Fig 5:**
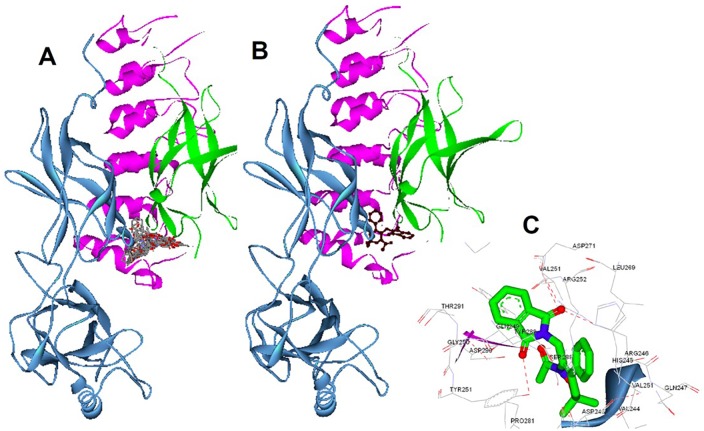
In silico interaction between the oxazines and IκBα/NF-κB complex. A. Representation of the native IκBα/NF-κB heterodimer and docked solution of tested oxazines (in stick representation). The sub-unit of p50 is represented in green, p65 in cyan, and IκBα in pink. B. Molecular docking of the lead structure API with the NF-κB heterodimer solution was shown. C. Interaction map lead compound API that bound with key amino acids of the crystal structure. Hydrogen bonding (black dots) between API oxygen atoms with Tyr251, and other Asp271, Arg246, His245 was shown.

Since 1,2-oxazines binds to NF-κB in vitro, we docked all the ligands to the IκBα/NF-κB crystal structure *via* multistep docking protocol implemented in the Accelrys Discovery Studio software. The docked poses were ranked based on their CDocker energy (kcal•mol^−1^) which was calculated and used as a mean for the binding strength of oxazines (Table 1). The tested oxazines were found to bind to the hydrophobic cavity that present between the interface of p65 and IκBα (Fig 5A). Consistent with bioactivity evaluation, the compound API interacted with the highest CDocker binding energy of -330.737 kcal•mol^−1^. Compound API (oxygen atom of pthalazine ring) makes hydrogen bonding with Tyr251, whereas the other oxygen forms strong hydrogen bond with Asp271, Arg246, and His245. Therefore, compound API scaffold bound strongly with hydrophobic Van der Waals interactions and a hydrogen bond to the backbone of many key amino acids of the NF-κB heterodimer (Fig 5B and 5C). Overall, most of the active oxazines biding energies are comparable to the compound BPO, which was previously identified as a selective inhibitor of NF-κB pathway.

### Modulatory effect of API on DSS-induced DAI, colon length and myeloperoxidase activity

**Fig 6:**
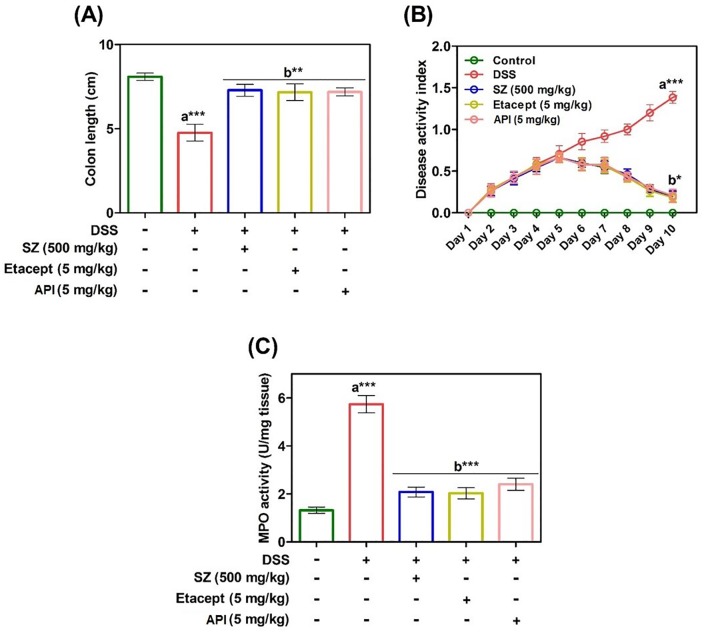
A. Extent of colonic inflammation was assessed indirectly by measuring the colon length of control and experimental mice. B. Severity of dextran sulphate sodium-induced colitis was monitored daily by assessing the DAI throughout the experimental period. From day 4 onward DSS induced a significant increase in DAI. C. Severity of colonic inflammation was assessed by colonic MPO activity from control and experimental mice. SZ-Sulfasalazine. Data are presented as mean ± S.E.M. * *p*<0.05; **p<0.01; ***p<0.001.

A selective novel low-molecular-weight inhibitor of IKKβ exhibits broad anti-inflammatory activity in various *in vivo* inflammatory models [40]. Given the relation with NF-κB inhibitors and inflammation, we assessed anti-inflammatory efficacy of API by determining the colon length, myeloperoxidase and DAI score in each group of DSS-induced colitis animals. In order to investigate the effect of API on colonic inflammation, colon length from caecum to anus of individual animals was measured. The colon length of DSS-induced animals found to be reduced significantly (*p*< 0.001) compared to RO control animals (Fig 6A). Whereas, the colon length of diseased animals treated with SZ, etacept and API showed significant increase (*p*< 0.05) in colon length compared to untreated diseased animals. Further evaluation revealed that, DAI score was increased in group 2 (DSS-induced) with respect to RO control animals (group 1) throughout the experimental period. Animals treated with etacept, SZ and API showed significantly decreased DAI score as compared to DSS-induced group. The DAI was found to be decreased from day 6 and reached to near normal at the end of the experimental period (Fig 6B). Additionally, MPO levels were estimated in order to assess the neutrophil infiltration and extent of colonic inflammation. From the results, it is clear that augmented levels of MPO in colon homogenates of diseased animals was observed compared to RO control animals (Fig 6C). In contrast, colon homogenates of API treated diseased animals exhibited decreased levels of MPO activity and it is similar to that of standard drugs used in the study (SZ and etacept).

### API reverses DSS-induced alteration of cytokines

**Fig 7:**
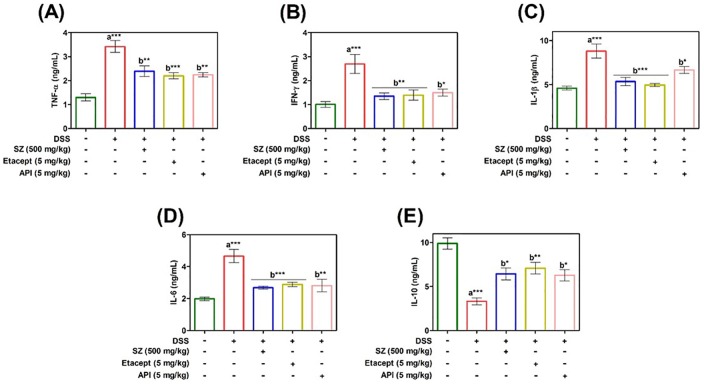
Serum cytokine profile of control and experimental mice were estimated using murine mini ELISA development kits according to the manufacturer’s protocol. A. TNF-α. B. IFN-γ. C. IL-1β. D. IL-6. and E. IL-10. SZ-Sulfasalazine. Data are presented as mean ± S.E.M. * *p*<0.05; **p< 0.01; ***p<0.001.

DSS-induced colitis model demonstrates an imbalance in the levels of serum pro- and anti-inflammatory cytokines. In order to confirm this notion, serum cytokine profiling was carried out using commercial ELISA kits. The pro-inflammatory cytokines such as TNF-α, IFN-γ, IL-6, and IL-1β were found to be significantly increased whereas, IL-10, an anti-inflammatory cytokine was found to be decreased in diseased animals in comparison with RO control animals. In contrast, Serum cytokine levels in API treated diseased animals were significantly reverted to basal levels. The results were well comparable to that of diseased animals treated with standard drugs, SZ and etacept (Fig 7A–7E).

### Modulatory effect of API on DSS-induced colonic histology

**Fig 8:**
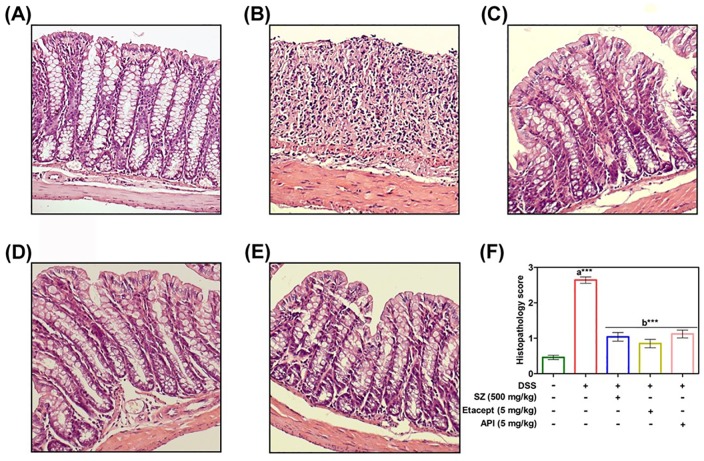
A. Represents colon section of RO control mice showing normal crypt structures along with intact mucosal and sub-mucosal epithelium. B. represents colon section of DSS-induced mice showing massive inflammation with complete loss of structural integrity of crypt epithelium. C-E. represents colons sections of DSS-induced animals treated with SZ, etacept and API respectively showing re-establishment of cryptic epithelial deterioration and with lesser infiltration of inflammatory cells (Magnification 200X). F. Represents the histology scoring of control and experimental animals. SZ-Sulfasalazine. Scoring is presented as mean ± S.E.M of five independent observations of colon sections. **p< 0.01; ***p<0.001. a is significant v/s RO water control and b is significant v/s DSS control.

DSS-induced colitis showed massive loss in the structural integrity of colon architecture as evidenced by histopathological analysis of colons by using H and E staining and as well as histopathology scoring (Fig 8A–8E). Colons of DSS-induced untreated animals were observed with substantial loss of cryptic epithelium, and infiltration of inflammatory leukocytes as compared to RO control (Fig 8F). While, colitis induced animals treated with SZ, etacept and API exhibited lesser infiltration of inflammatory leukocytes along with reversal of normal architecture of colon in comparison with untreated animals.

## Discussion

NF-κB is an inducible transcription factor present in the cytoplasm of most mammalian cells [8]. Researchers have rightly called NF-κB as a double-edged sword due to its entanglement in the proper functioning of immune system and its inappropriate activation may result inflammation and tumorigenesis [41]. Aberrant activation of NF-κB has been detected and reported in several types of human malignancies [42]. Several natural and synthetic small molecules with the wide-array of heterocyclic nucleus including oxazines, coumarins, biscoumarins, benzofurans (rocaglamide), pyridine conjugates (sulfasalazine), pyrans (trichodion) and non-steroidal anti-inflammatory drugs such as sodium salicylate, ibuprofen, sulindac and indomethacin have been evaluated for their inhibitory activity against NF-κB signaling pathway [43] and many of them have been unsuccessful in advancement to clinics due to their limited efficacy. Among these scaffolds, we were interested to prepare the oxazine derivatives, because of their good bioavailability and acceptable safety profile in preclinical studies on rat and cynomolgus monkey toxicity studies [44]. Furthermore, pharmacodynamics and bioavailability of bemoradan (a benzoxazine derivative) in humans, rats and dogs demonstrated the rapid absorption after oral dosing, linear pharmacodynamics and long elimination half-lives across species [45].

Therefore, the present study focusses on synthesis of a panel of oxazines, the determination of lead compound (API), demonstration of mechanism of lead compound as it targets NF-κB in vitro and in vivo, and validation of the target using in silico analysis. The antiproliferative efficacy exhibited by oxazines are in agreement with previous studies against lung and colon cancer [12], hepatocellular carcinoma [13], breast cancer [15] and pheochromocytoma cells [16]. Although oxazine derivatives have been presented as NF-κB inhibitors in several types of cancer cells, to the best of our knowledge, this is the first time, we are demonstrating the inhibitory efficacy of 1,2-oxazines against NF-κB in colitis-induced mice model. Increased activation of NF-κB has been reported to be involved in regulation of inflammatory response in IBD [46,47], which makes it most likely that oxazines induce their inhibitory effect on animals affected with IBD by suppressing NF-κB.

NF-κB has a crucial role in regulation of inflammatory responses, innate immunity, cell proliferation and apoptosis and it has been identified as a key factor in cancer initiation and progression [48]. The role of NF-κB in regulation of immune response and its persistent activation in many types of cancers to exert its pro-tumorigenic effect increases the complexity in designing of inhibitors against NF-κB. However, combinations of NF-κB inhibitors along with classical chemotherapeutics have been reported to produce promising synergetic effects [48–52]. Therefore, we opted colitis induced mice model to study the possible inhibitory effect of oxazines against NF-κB. The histological analysis of colon clearly demonstrated the significant restoration of cryptic epithelium architecture and decrease in the myeloperoxidase activity in colonic extract and disease activity index, which is highly comparable with Etacept and Sulfasalazine. The increased anti-inflammatory cytokines and decreased pro-inflammatory cytokines in the API-treated group providing a better in-sight into the mode of action of oxazines.

## Conclusion

In summary, herein we synthesized a series of 1,2-oxazines and screened for their antiproliferative activity against colon cancer cell lines and identified the bioactive compound. Considering the inhibitory efficacy of oxazine derivatives NF-κB signaling cascade, we tested the lead compounds against possible blockade of NF-κB pathway and found significant reduction in NF-κB DNA binding ability and NF-κB-dependent luciferase expression. Based on NF-κB entanglement with inflammation, we established the DSS-induced IBD model and presented the anti-inflammatory activity of the lead compound. The *in vitro* and *in vivo* results were validated using molecular docking analysis. In conclusion, this report proposes 1,2-oxazines as therapeutically potential scaffolds to develop drugs against inflammatory diseases.
