# Correction: Novel Synthetic Oxazines Target NF-κB in Colon Cancer *In Vitro* and Inflammatory Bowel Disease *In Vivo*

**DOI:** 10.1371/journal.pone.0175659

**Published:** 2017-04-06

**Authors:** Anilkumar C. Nirvanappa, Chakrabhavi Dhananjaya Mohan, Shobith Rangappa, Hanumappa Ananda, Alexey Yu Sukhorukov, Muthu K. Shanmugam, Mahalingam S. Sundaram, Siddaiah Chandra Nayaka, Kesturu S. Girish, Arunachalam Chinnathambi, M. E. Zayed, Sulaiman Ali Alharbi, Gautam Sethi, Basappa, Kanchugarakoppal S. Rangappa

Fig 2 appears incorrectly in the published article. Please see the correct Fig 2 and its caption here.

**Fig 1:**
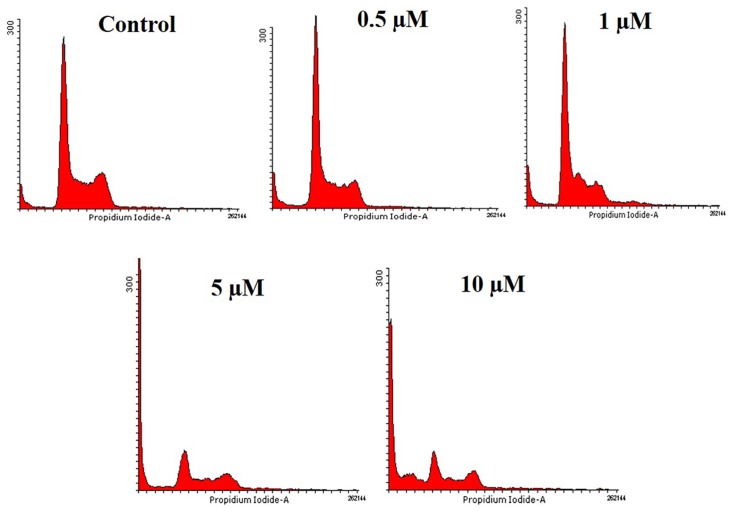
HCT116 cells were treated with different doses of API (0.5, 1, 5, and 10 μM) for 48 h, harvested and stained with propidium iodide and subjected to flow cytometry. Histogram obtained indicated the accumulation cells in sub-G1 phase.
